# Staring at the onco-exaptation: the two-faced medley of an ancient retrovirus, *HERVH*

**DOI:** 10.1172/JCI172278

**Published:** 2023-07-17

**Authors:** Manvendra Singh, Aleksandra M. Kondraskhina, Laurence D. Hurst, Zsuzsanna Izsvák

**Affiliations:** 1 Max Planck Institute of Multidisciplinary Sciences, City Campus, Göttingen, Germany.; 2 Max-Delbrück-Center for Molecular Medicine in the Helmholtz Society, Berlin, Germany.; 3 The Milner Centre for Evolution, Department of Biology and Biochemistry, University of Bath, Bath, United Kingdom.

## Abstract

Cell senescence suppresses tumors by arresting cells at risk of becoming malignant. However, this process in turn can affect the microenvironment, leading to acquisition of a senescence-associated secretory phenotype (SASP) that renders senescent cells proinflammatory and results in tumor progression. But how is SASP controlled? In this issue of the *JCI*, Attig and Pape et al. describe the role of chimeric calbindin 1 (*CALB1*) transcripts, which are driven by an upstream human endogenous retrovirus subfamily H (*HERVH*) element. The authors propose that in lung squamous cell carcinoma (LUSC), HERVH-driven isoforms of calbindin (*HERVH-CALB1*) counteract SASP. As an alternative promoter, *HERVH* drove calbindin isoforms that prevented cancer cell senescence and associated inflammation, which was associated with better patient survival. We comment on the similarities between HERVH-CALB1–related cellular fitness in cancer and early embryogenesis and discuss the potential benefits of *HERVH-*driven chimeric transcripts.

## Communication between cancer cells and antitumor immune responses

Escaping senescence is thought to be a necessary step in cancer initiation (1). However, senescence is a double-edged sword, as this cellular stage is associated with the senescence-associated secretory phenotype (SASP), characterized by activated chemokine-signaling pathways (2) that correlate with neutrophil infiltration and worse prognosis. Lung squamous cell carcinoma (LUSC) is a type of lung cancer that persists globally among the main causes of cancer-related death in the world. The classical therapy for LUSC patients includes surgical removal of carcinomas, followed by radiotherapy and/or chemotherapy (3). A more personalized therapy utilizes a tailored approach by inhibiting molecular pathways that target patient-specific driver mutations, which in LUSC are restricted to only two oncogenes (*NTRK*, *MET*), whereas in other lung cancers, a larger number of target oncogenes are known (3). Recently, immune checkpoint inhibitors (ICIs) have been used to activate the immune systems of patients to destroy tumor cells (4). Despite the considerable potential of immune treatments, only approximately 20% of patients respond to them (3), urging a better understanding of cellular heterogeneity and communication between cancer cells and antitumor immune responses.

In this issue of the *JCI*, Attig, Pape, and authors started by analyzing the LUSC cohort in The Cancer Genome Atlas (TCGA) database, which showed a correlation between the expression of a human endogenous retroviral (HERV) locus and better patient survival (5).

## Onco-exaptation

About half of the human genome derives from endogenous retroelements (EREs), including the remnants of previous retroviral infections (HERVs) that occupy around 8% of the genome. Elevated transcription of EREs has been frequently reported from various pathologies (6–8); however, their potential contribution to disease progression is not well understood. While retrotransposed EREs (e.g., LINE-1, SVA, Alu) can be directly detected in rearranged oncogenes (6), EREs could also cause pathology through retrotransposition-independent mechanisms. Indeed, ERE-derived transcripts might trigger the innate immune response and contribute to inflammation, various degenerative diseases, and cancer (7–11). In another scenario, transcriptional control of a HERV-derived promoter embedded in a long terminal repeat (LTR) drives expression of neighboring oncogenes and contribute to oncogenesis; the process has been termed “onco-exaptation” (12). Several onco-exaptation events have already been validated in various HERV families that form chimeric transcripts with their neighboring genes in different cancer types (12–14). Typically, these onco-exaptation events are characterized by LTR-HERV–enforced upregulation of the neighboring oncogene and are associated with worsened patient survival.

## HERVH as an alternative promoter for CALB1 transcripts

**Figure 1:**
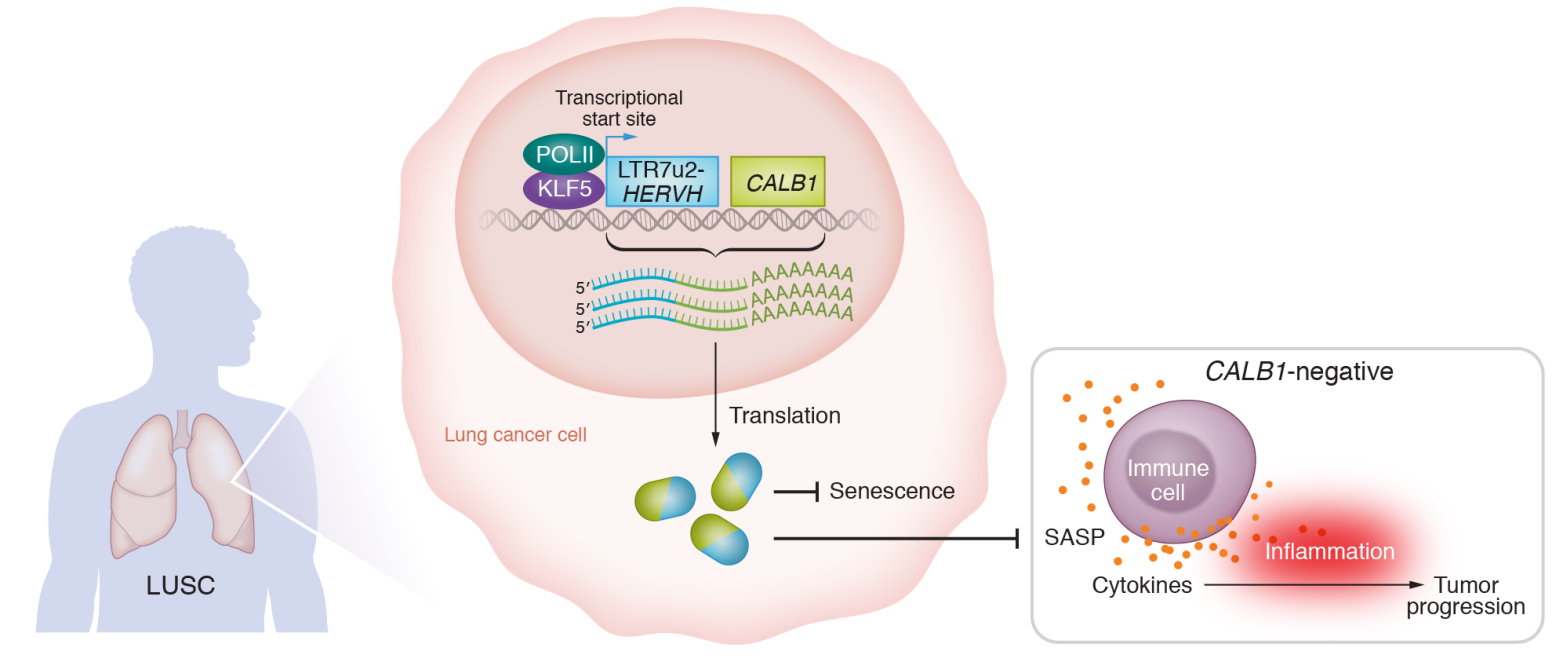
HERVH-driven CALB1 isoforms counteract senescence and associated inflammatory response in LUSC tumors. KLF5-responsive LTR7u2-*HERVH* generates three variants of *HERVH-CALB1* chimeric transcripts in LUSC. The translated protein products inhibit senescence and SASP, thus attenuating inflammatory tumor progression. In contrast, CALB1-negative cancer cells show a SASP phenotype, produce high levels of CXCL8, and display protumor inflammation.

Attig, Pape, and coauthors used bulk and single-cell transcriptomic data overlaid with genetic and biochemical functional experiments to characterize a chimeric transcript production event that was enforced by a copy of HERV subfamily H (*HERVH*) at the calbindin 1 (*CALB1*) locus in LUSC (5). Although the event is referred to as “onco-exaptation,” it is not a typical example. First, CALB1 is not a known oncogene (although it inhibits senescence in ovarian cancer cells) (15); second, increased expression from the locus correlates with better (rather than worse) patient survival. CALB1 is a member of the calmodulin family and buffers calcium entry into cells upon stimulation of glutamate receptors (16). Recently, calcium ions have been revealed as regulators of cellular senescence, and importantly, CALB1 likely regulates such ions in senescent cells (17). Attig, Pape, et al. report that, although undetectable in healthy airways, three *HERVH-CALB1* chimeric transcripts from LUSC have levels that increase gradually in preinvasive airway lesions (5). The effect of *HERVH-CALB1* expression was mediated by the encoded protein(s). *HERVH* generated three variants of highly expressed chimeric transcripts that were translated into three N-terminal truncated, but identical, CALB1 proteins (5). These CALB1 isoforms antagonized senescence and promoted cellular fitness (5), most likely in a way similar to that of canonical calbindin (16). Using loss-of-function experiments, Attig, Pape, and colleagues (5) demonstrated that CALB1 played a role in regulating CXCL8 secretion as part of SASP. The truncated HERVH-CALB1 protein isoform and full-length CALB1 were functionally equivalent in controlling CXCL8 release. Therefore, Attig, Pape, and authors suggested that the cooption of HERVH was restricted to the provision of alternative promoter activity, controlled by KLF5. Located upstream of *CALB1*, the *HERVH* element generated three CALB1 isoforms that counteracted senescence and associated inflammation. Thus, *HERVH-CALB1–*encoded isoforms specifically modulated crosstalk between cancer cells and antitumor immune responses by attenuating the inflammatory response in LUSC (Figure 1). The findings (5) also suggest that variability in *HERVH-CALB1* expression might be associated with cellular heterogeneity observed in LUSC tumors.

Previously *HERVH* was shown to generate chimeric RNA products, e.g., long noncoding RNAs (lncRNAs) (18). Attig, Pape, et al. (5) report that the effect of *HERVH-CALB1* expression was mediated by the encoded protein(s). By buffering intracellular Ca^2+^ levels, CALB1 antagonizes senescence and promotes the replicative capacity of cells (16). Using loss-of-function experiments, the authors demonstrated that CALB1 played a role in regulating CXCL8 secretion as part of SASP. The HERVH-CALB1 protein isoform and full-length CALB1 were functionally equivalent in controlling CXCL8 release. Attig, Pape, and authors, therefore, suggested that the cooption of HERVH was restricted to the provision of alternative promoter activity. Although there is currently no evidence to support this hypothesis, potentially altered function of the truncated HERVH-driven CALB1 isoform may have importance, as the oncogenic properties of canonical CALB1 have been reported (15).

## Analogy between embryonic development and tumorigenesis

The canonical CALB1 is most abundantly expressed in neuronal cells. A chimeric *HERVH-CALB1* transcript is also expressed in healthy neurons and has also been reported in human embryonic stem (ES) cells and in the epiblast of preimplantation embryos (18, 19), suggesting that the chimeric transcript might represent a beneficial, yet to be characterized, exaptation event. Therefore, Attig, Pape, et al. (5) compels us to look back into early embryonic development, where pluripotency exists. Intriguingly, the LTR7 *HERVH* family is exceptional among EREs (20) as being coopted for early human embryonic development, supporting self-renewal and maintaining the pluripotent state; and its expression is postulated as a marker for human pluripotency (18).

Indeed, there is probably a profound analogy between embryonic development and tumorigenesis at the level of biological processes (21). Speaking of ES cell self-renewal, one can discern similar events also driving tumorigenesis (22). Some of the core pioneer transcription factors that control the fate of the early embryo are also activated in certain tumor cells. It is quite possible that they regulate shared biological processes between embryonic and cancer cells, such as self-renewal. It is even possible that HERVH regulatory activities impinge on the vital cellular processes of embryonic and cancer cells, thus coopting HERVH activity for host fitness in two different niches.

## HERVH-CALB1 is expressed in the pluripotent epiblast

Attig and Pape et al. reveal that HERVH-CALB1 expression is driven by KLF5 (5), in contrast to KLF4, which drives LTR7-*HERVH* transcripts (18). In fact, HERVH-CALB1 expression is driven by a recently classified LTR7u2 (23), which unlike LTR7, has a KLF5-binding site and is expressed in a slightly different niche. *HERVH-CALB1*, driven by LTR7u2, is specifically expressed in the pluripotent epiblast (5, 19) and may play a functional role, particularly at this stage, in contrast to LTR7, in primed pluripotency (18). So why does KLF4, a pioneer factor that regulates LTR7 *HERVH* in primed pluripotency, not control LTR7-*HERVH* expression in cancer cells? Although the answer is not yet clear, one relevant observation relates to chromatin accessibility. KLF5 may render chromatin at *HERVH* loci more accessible to other transcription factors, as this pattern has been seen in naive ES cell cultures (24).

Future work might explore the molecular criteria that underpin the differential activity of LTR7 in different cellular niches. It is tempting to speculate as to whether these pioneer factors compete to occupy the LTR7-*HERVH* loci, whether a set of cofactors influences their recruitment, or whether an interplay between repressive and pioneer factors precisely determines the heterogenous nature of HERVH activity.

## Conclusions and future directions

EREs are emerging as promising therapeutic targets in cancer. ERE-mediated activation of the innate immune response (termed “viral mimicry”) with subsequent inflammation has been suggested as a mechanism for sensitizing tumors to immunotherapy (9). While modulation of ERE expression has been addressed in preclinical and clinical strategies to treat cancer, a caution is warranted because the mechanisms by which EREs contribute to pathologies are not well understood. For example, the attenuating effect of the *HERVH*-driven CALB1 on inflammation has the opposite (5) effect of that seen when phylogenetically young (less than seven million year) EREs are overexpressed (10, 11). Curiously, other phylogenetically young EREs that also show opposing collective actions have also been observed in the early embryo (25).

## Version 1 07/17/2023

Electronic publication
